# Complete Genome Sequence of the Model Rhizosphere Strain *Azospirillum brasilense* Az39, Successfully Applied in Agriculture

**DOI:** 10.1128/genomeA.00683-14

**Published:** 2014-07-24

**Authors:** Diego Rivera, Santiago Revale, Romina Molina, José Gualpa, Mariana Puente, Guillermo Maroniche, Gastón Paris, David Baker, Bernardo Clavijo, Kirsten McLay, Stijn Spaepen, Alejandro Perticari, Martín Vazquez, Florence Wisniewski-Dyé, Chris Watkins, Francisco Martínez-Abarca, Jos Vanderleyden, Fabricio Cassán

**Affiliations:** a Universidad Nacional de Río Cuarto (UNRC), Córdoba, Argentina; b Instituto de Agrobiotecnología Rosario (INDEAR), Rosario, Argentina; c Instituto de Microbiología y Zoología Agrícola, (IMYZA-INTA), Castelar, Buenos Aires, Argentina; d Instituto Leloir, Buenos Aires, Argentina; e The Genome Analysis Centre (TGAC), Norwich, United Kingdom; f Katholieke Universiteit Leuven, Leuven, Belgium; g Max Planck Institute for Plant Breeding Research, Cologne, Germany; h Ecologie Microbienne, Université Lyon, Villeurbanne, France; i Grupo de Ecología Genética de la Rizósfera, Estación Experimental del Zaidín (CSIC), Granada, Spain

## Abstract

We present the complete genome sequence of *Azospirillum brasilense* Az39, isolated from wheat roots in the central region of Argentina and used as inoculant in extensive and intensive agriculture during the last four decades. The genome consists of 7.39 Mb, distributed in six replicons: one chromosome, three chromids, and two plasmids.

## GENOME ANNOUNCEMENT

*Azospirillum* sp. is one of the best-studied plant-growth-promoting rhizobacteria at present. Members of this genus colonize more than 100 plant species and significantly improve their growth and productivity under field conditions (1). One of the main characteristics of *Azospirillum* sp. proposed to explain plant growth promotion has been related to its ability to produce plant growth regulators as auxins, cytokinins, gibberellins, ethylene, abscisic acid, nitric oxide, and polyamines (2–8). *Azospirillum brasilense* Az39 was isolated in 1982 from surface-sterilized wheat seedlings in Marcos Juarez, Argentina, and selected for inoculant formulation based on its ability to increase crop yields of maize and wheat under agronomic conditions (9). The potential mechanisms responsible for growth promotion in strain Az39 have been partially unraveled (10–13).

The genome sequence was obtained using a combined whole-genome shotgun and 8-kb paired-end strategy with a 454 GS FLX Titanium pyrosequencer at INDEAR (Argentina), resulting in a 21-fold genome coverage. Sequencing reads were *de novo* assembled (Newbler v 2.8), resulting in 6 scaffolds (>160 kbp each; *N*_50_, 1,908,534 bp). The closure of the gap intra- and interscaffolds was achieved by detailed observation of relevant sequencing reads using the Geneious R7 software platform (14). Optical mapping analysis was performed with an OpGen Argus optical mapper at TGAC (United Kingdom) to validate the final assembly. In agreement with the bioinformatic data, pulsed-field gel electrophoresis (PFGE) analysis of total DNA revealed the presence of six replicons in *A. brasilense* Az39, defined as one chromosome, three chromids, and two plasmids. The presence of six to seven replicons is a common feature of *Azospirillum* genomes (15–17). Replicon sizes and their G+C contents were 3,064,393 bp (68.4%) for the chromosome; 1,901,707 bp (68.4%), 933,960 bp (68.6%), and 641,573 bp (69.2%) for the chromids (AbAZ39_p1, AbAZ39_p2, and AbAZ39_p4); and 686,487 bp (69.5%) and 163,159 bp (65.6%) for the plasmids (AbAZ39_p3 and AbAZ39_p5).

Genome annotation was done using the NCBI Prokaryotic Genomes Automatic Annotation Pipeline (PGAAP) (18). The complete genome consists of 6,311 protein-coding sequences (2,763 on the chromosome, 1,605 on AbAZ39_p1, 744 on AbAZ39_p2, 534 on AbAZ39_p3, 557 on AbAZ39_p4, and 108 on AbAZ39_p5). Similarly to other species of the *Azospirillum* genera, Az39 contains multiple ribosomal operons at different replicons (15–17). Eight rRNA operons are complete and one lacks the 5S rRNA subunit. Complete operons are distributed with two in the chromosome, four in AbAZ39_p1, and two in AbAZ39_p4, while the incomplete one is located on the chromid AbAZ39_p2. Eighty-seven tRNA loci (distributed 44 on the chromosome, 42 on the chromids, and 1 on the plasmids) were identified. The putative genes involved in plant growth promotion mechanisms of Az39 were determined by the use of the RAST annotation server (19) and KAAS (20).

The *A. brasilense* Az39 genome contains genes related to nitrogen fixation; phytohormones and plant growth regulators biosynthesis; biofilms formation and type I, II, and VI secretion systems. The genome sequence of Az39 provides a genomic basis for in-depth comparative genome analyses, to elucidate the specific mechanisms of *Azospirillum*-plant interactions.

### Nucleotide sequence accession numbers.

The complete genome sequence of *Azospirillum brasilense* Az39 is available at NCBI GenBank under the accession numbers CP007793 for the chromosome and CP007794 to CP007798 for the other replicons.

## References

[1] Bashan Y de-Bashan L 2010 How the plant growth-promoting bacterium *Azospirillum* promotes plant growth—a critical assessment. Advances Agron. 108:77–136. 10.1016/S0065-2113(10)08002-8

[2] Prinsen E Costacurta A Michiels K Vanderleyden J Van Onckelen H 1993 *Azospirillum brasilense* indole-3-acetic acid biosynthesis: evidence for a non-tryptophan dependent pathway. Mol. Plant. Microb. Interact. 6:609–615

[3] Tien TM Gaskins MH Hubbell DH 1979 Plant growth substances produced by *Azospirillum brasilense* and their effect on the growth of pearl millet (*Pennisetum americanum* L). Appl. Environ. Microbiol. 37:1016–1024 16345372 10.1128/aem.37.5.1016-1024.1979 PMC243341

[4] Bottini R Fulchieri M Pearce D Pharis RP 1989 Identification of gibberellins A_1_, A_3_, and Iso-A_3_ in cultures of *Azospirillum lipoferum*. Plant Physiol. 90:45–47. 10.1104/pp.90.1.45 16666766 PMC1061674

[5] Strzelczyk E Kamper M Li C 1994 Cytocinin-like-substances and ethylene production by *Azospirillum* in media with different carbon sources. Microbiol. Res. 149:55–60. 10.1016/S0944-5013(11)80136-9

[6] Cohen A Bottini R Piccoli P 2008 *Azospirillum brasilense* sp. 245 produces ABA in chemically defined culture medium and increases ABA content in Arabidopsis plants. Plant Growth Regul. 54:97–103. 10.1007/s10725-007-9232-9

[7] Creus CM Graziano M Casanovas EM Pereyra MA Simontacchi M Puntarulo S Barassi CA Lamattina L 2005 Nitric oxide is involved in the *Azospirillum brasilense*-induced lateral root formation in tomato. Planta 221:297–303. 10.1007/s00425-005-1523-7 15824907

[8] Cassán F Maiale S Masciarelli O Vidal A Luna V Ruiz O 2009 Cadaverine production by *Azospirillum brasilense* and its possible role in plant growth promotion and osmotic stress mitigation. Eur. J. Soil Biol. 45:12–19. 10.1016/j.ejsobi.2008.08.003

[9] Díaz-Zorita M Fernández Canigia M 2009 Field performance of a liquid formulation of *Azospirillum brasilense* on dryland wheat productivity. Eur. J. Soil Biol. 45:3–11. 10.1016/j.ejsobi.2008.07.001

[10] Perrig D Boiero L Masciarelli OA Penna C Ruiz OA Cassán FD Luna MV 2007 Plant-growth-promoting compounds produced by two agronomically important strains of *Azospirillum brasilense*, and their implications for inoculant formulation. Appl. Microbiol. Biotechnol. 75:1143–1150. 10.1007/s00253-007-0909-9 17345081

[11] Cassán F Perrig D Sgroy V Masciarelli O Penna C Luna V 2009 *Azospirillum brasilense* Az39 and *Bradyrhizobium japonicum* E 109 promote seed germination and early seedling growth, independently or co-inoculated in maize (*Zea mays* L.) and soybean (*Glycine max* L.). Eur. J. Soil Biol. 45:28–35. 10.1016/j.ejsobi.2008.08.005

[12] Cassán F Spaepen S Vanderleyden J 2010 Abstract. Indole-3-acetic acid biosynthesis by *Azospirillum brasilense* Az39 and its regulation under biotic and abiotic stress conditions, p 85 20th International Conference on Plant Growth Substances.

[13] Rodríguez Cáceres E Di Ciocco A César A Carletti S 2008 25 Editorial Años de investigación de *Azospirillum brasilense* Az39 en Argentina, p 179–188 *In* Cassán F Salamone I (ed), Azospirillum sp.: cell physiology, plant interactions and agronomic research in Argentina. Asociación Argentina de Microbiología, Buenos Aires, Argentina

[14] Kearse M Moir R Wilson A Stones-Havas S Cheung M Sturrock S Buxton S Cooper A Markowitz S Duran C Thierer T Ashton B Meintjes P Drummond A 2012 Geneious basic: an integrated and extendable desktop software platform for the organization and analysis of sequence data. Bioinformatics 28:1647–1649. 10.1093/bioinformatics/bts199 22543367 PMC3371832

[15] Martin-Didonet CC Chubatsu LS Souza EM Kleina M Rego FG Rigo LU Yates MG Pedrosa FO 2000 Genome structure of the genus *Azospirillum*. J. Bacteriol. 182:4113–4116. 10.1128/JB.182.14.4113-4116.2000 10869094 PMC94601

[16] Wisniewski-Dyé F Borziak K Khalsa-Moyers G Alexandre G Sukharnikov LO Wuichet K Husrt GB McDonald WH Robertson JS Barbe V Calteau A Rouy Z Mangenot S Prigent-Combaret C Normand P Boyer M Siguier P Dessaux Y Elmerich C Condemine G Krishnen G Kennedy I Paterson AH González V Mavingui P Zhulin IB 2011 *Azospirillum* genomes reveal transition of bacteria from aquatic to terrestrial environments. PLOS Genet. 7 e1002430. 10.1371/journal.pgen.1002430 22216014 PMC3245306

[17] Wisniewski-Dyé F Lozano L Acosta-Cruz E Borland S Drogue B Prigent-Combaret C Rouy Z Barbe V Mendoza Herrera AM González V Mavingui P 2012 Genome sequence of *Azospirillum brasilense* CBG497 and comparative analyses of *Azospirillum* core and accessory genomes provide insight into niche adaptation. Genes (Basel) 3:576–602. 10.3390/genes3040576 24705077 PMC3899980

[18] Angiuoli SV Gussman A Klimke W Cochrane G Field D Garrity G Kodira CD Kyrpides N Madupu R Markowitz V Tatusova T Thomson N White O 2008 Toward an online repository of Standard Operating Procedures (SOPs) for (meta)genomic annotation. Omics 12:137–141. 10.1089/omi.2008.0017 18416670 PMC3196215

[19] Aziz RK Bartels D Best AA DeJongh M Disz T Edwards RA Formsma K Gerdes S Glass EM Kubal M Meyer F Olsen GJ Olson R Osterman AL Overbeek RA McNeil LK Paarmann D Paczian T Parrello B Pusch GD Reich C Stevens R Vassieva O Vonstein V Wilke A Zagnitko O 2008 The RAST server: rapid annotations using subsystems technology. BMC Genomics 9:75. 10.1186/1471-2164-9-75 18261238 PMC2265698

[20] Moriya Y Itoh M Okuda S Yoshizawa AC Kanehisa M 2007 KAAS: an automatic genome annotation and pathway reconstruction server. Nucleic Acids Res. 35:W182–W185. 10.1093/nar/gkm321 17526522 PMC1933193

